# E3 Ubiquitin Ligases as Immunotherapeutic Target in Atherosclerotic Cardiovascular Disease

**DOI:** 10.3389/fcvm.2020.00106

**Published:** 2020-06-05

**Authors:** Kikkie Poels, Winnie G. Vos, Esther Lutgens, Tom T. P. Seijkens

**Affiliations:** ^1^Department of Medical Biochemistry, Amsterdam Cardiovascular Sciences (ACS), Amsterdam UMC, Location AMC, University of Amsterdam, Amsterdam, Netherlands; ^2^Institute for Cardiovascular Prevention (IPEK), Ludwig Maximilian's University, Munich, Germany; ^3^German Centre for Cardiovascular Research (DZHK), Partner Site Munich Heart Alliance, Munich, Germany; ^4^Department of Internal Medicine, Amsterdam UMC, Location VUmc, VU University, Amsterdam, Netherlands; ^5^Department of Hematology, Amsterdam UMC, Location VUmc, VU University, Amsterdam, Netherlands

**Keywords:** atherosclerosis, inflammation, E3 ubiquitin ligases, Cbl-B, Itch, GRAIL

## Abstract

Chronic low-grade inflammation drives atherosclerosis and despite optimal pharmacological treatment of classical cardiovascular risk factors, one third of the patients with atherosclerotic cardiovascular disease has elevated inflammatory biomarkers. Additional anti-inflammatory strategies to target this residual inflammatory cardiovascular risk are therefore required. T-cells are a dominant cell type in human atherosclerotic lesions. Modulation of T-cell activation is therefore a potential strategy to target inflammation in atherosclerosis. Ubiquitination is an important regulatory mechanism of T-cell activation and several E3 ubiquitin ligases, including casitas B-lineage lymphoma proto-oncogene B (Cbl-B), itchy homolog (Itch), and gene related to anergy in lymphocytes (GRAIL), function as a natural brake on T-cell activation. In this review we discuss recent insights on the role of Cbl-B, Itch, and GRAIL in atherosclerosis and explore the therapeutic potential of these E3 ubiquitin ligases in cardiovascular medicine.

## Introduction

Atherosclerosis, a chronic lipid-driven inflammatory disease of the large arteries, is a major underlying cause of cardiovascular diseases (CVD) (1). Despite optimal primary and secondary pharmacological prevention by lipid lowering therapies and anti-platelet drugs, a significant part of the population develops atherosclerotic CVD, suggesting that additional factors drive atherogenesis in these subjects (2). One third of the patients with stable coronary artery disease and on-target cholesterol levels (<70 mg/dl) have elevated levels of the inflammatory biomarker high-sensitivity C-reactive protein (hsCRP), indicating that inflammation is an independent risk factor for atherosclerotic CVD that is not targeted by current pharmacological interventions (3).

The therapeutic potential of anti-inflammatory strategies in cardiovascular medicine is highlighted by several clinical trials (4–7). For example, the Canakinumab Anti-inflammatory Thrombosis Outcome Study (CANTOS) trial demonstrated that antibody-mediated inhibition of IL1β reduced recurrent CVD in patients with elevated hsCRP levels (>2 mg/l) (4). In the Colchicine Cardiovascular Outcomes Trial (COLCOT), colchicine reduced a composite endpoint of cardiovascular death and various presentations of recurrent atherosclerotic CVD among patients with a recent myocardial infarction (5). In contrast, the Cardiovascular Inflammation Reduction Trial (CIRT) failed to show beneficial cardiovascular effects of methotrexate in patients with type 2 diabetes or the metabolic syndrome and a history of CVD (6). Importantly, patients in CIRT had median hsCRP levels of 1.6 mg/l, reflecting a lower residual inflammatory risk in comparison to patients in CANTOS, who had median hsCRP levels of 4.2 mg/l (4, 6). These landmark trials emphasize that anti-inflammatory interventions have the potential to reduce (recurrent) atherosclerotic CVD in patients with a substantial residual inflammatory risk, which fuels the search for novel immunomodulatory strategies to temper inflammation in atherosclerosis.

Recent studies demonstrated that T-cells are a dominant cell type in human atherosclerotic lesions and comprise 65% of the immune cell content (8). Transcriptional and flow cytometric analyses of human plaques demonstrated that both CD4^+^ helper T-cells and CD8^+^ cytotoxic T-cells have an activated profile, especially in patients with symptomatic CVD, which highlights the importance of these cells in atherosclerosis (8, 9). In accordance with many preclinical studies, these findings indicate that modulation of T-cell activation is an attractive strategy to temper inflammation in atherosclerosis (1, 10, 11). The three-step process of T-cell activation is initiated by ligation of the T-cell receptor (TCR) and followed by a second signal that is provided by immune checkpoint proteins (10). Co-stimulatory and co-inhibitory molecules, which are expressed on antigen presenting cells, are the predominant members of the immune checkpoint protein family and may either enhance or hamper T-cell activation (10). The third signal is provided by soluble factors, such as cytokines (10). In the past two decades, the E3 ubiquitin ligases casitas B-lineage lymphoma proto-oncogene B (Cbl-B), itchy homolog (Itch), and gene related to anergy in lymphocytes (GRAIL) have emerged as key regulators of T-cell activation (12–15). Here, we discuss the role of these E3 ubiquitin ligases in atherosclerosis and explore their therapeutic potential in cardiovascular medicine.

## E3 Ubiquitin Ligases

**Figure 1:**
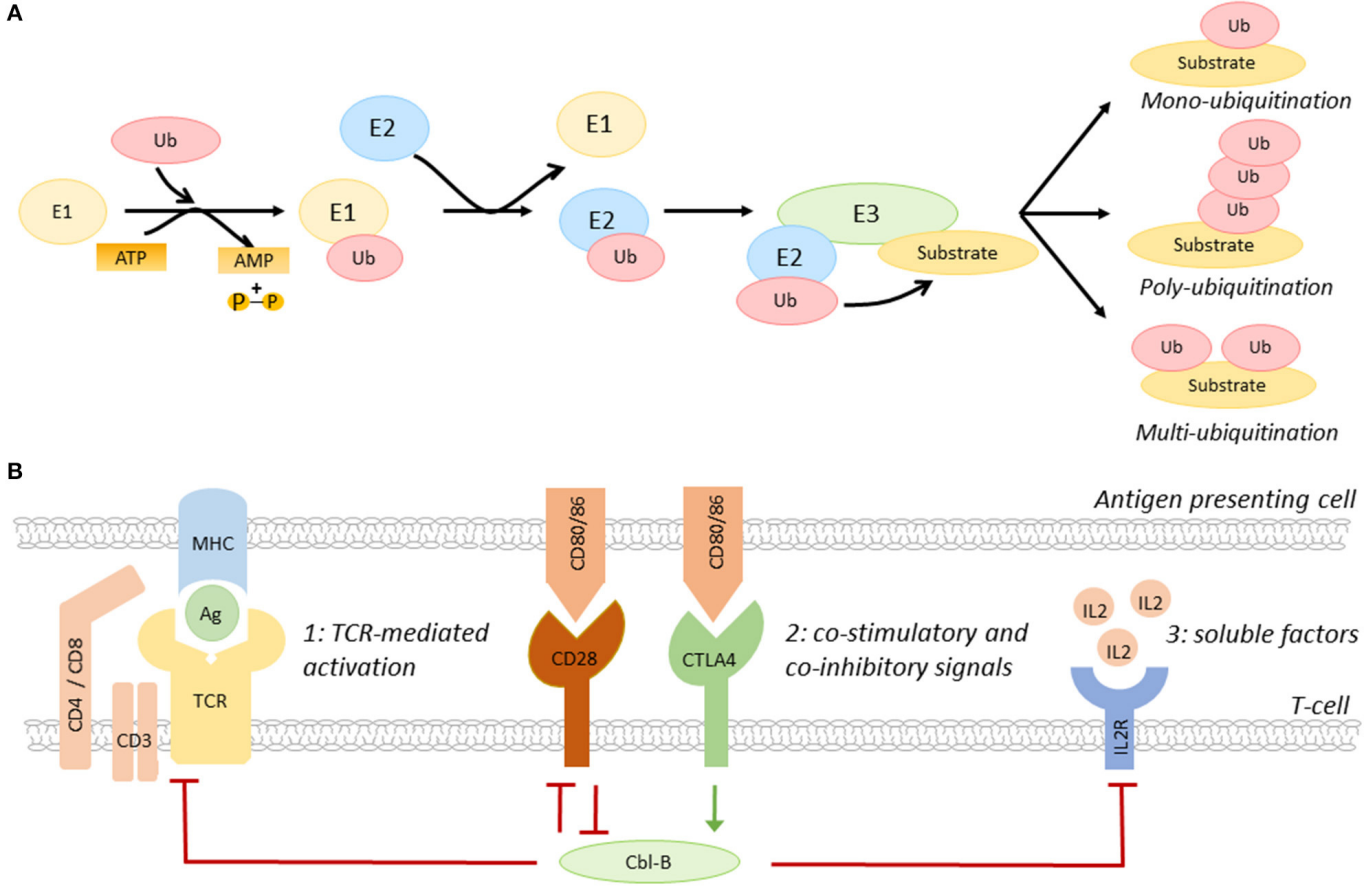
The role of Cbl-B in T-cell activation. **(A)** Schematic overview of the ubiquitination pathway. **(B)** Co-inhibitory molecules, such as CTLA4, induce Cbl-B expression, whereas the co-stimulatory molecule CD28 limits the expression of this E3 ubiquitin ligase. Cbl-B negatively regulates the three steps of T-cell activation, including T-cell receptor expression and signaling (1), immune checkpoint protein-induced signaling (2), and the production of IL2 and IL2R-induced signaling pathways (3). IL2R, IL2 receptor; Ag, antigen; MHC, major histocompatibility complex.

Ubiquitination, the post-translational process that results in the conjugation of the peptide ubiquitin to a lysine residue on a substrate protein, is a key regulatory mechanisms of many biological processes, including immune cell activation, as recently reviewed by Tang and colleagues (15). Ubiquitination affects the function, cellular localization, and/or degradation of target proteins and the functional outcome depends on the generated ubiquitin signal, e.g., mono- or poly-ubiquitination (13–15). Ubiquitination starts with the conjugation of ubiquitin to an E1 ubiquitin activating enzyme, then, ubiquitin is transferred to an E2 ubiquitin conjugating enzyme (13–15). Subsequently, the ubiquitin-E2 complex interacts with an E3 ubiquitin ligase, resulting in a covalent bond between ubiquitin and the target protein (Figure 1A) (13–15).

E3 ubiquitin ligases are a family of >600 proteins and determine substrate specificity of the ubiquitination process (13–15). Three types of E3 ubiquitin ligases are distinguished; really interesting new gene (RING) E3 ligases, homologous to E6-associated protein carboxyl terminus (HECT) E3 ligases, and RING-between-RING (RBR) ligases (13–15). Whereas RING ligases function as scaffold for E2 ligases and the target, HECT E3 ligases have a catalytic function that facilitates the transfer of ubiquitin to the target protein, and RBR ligases use a hybrid mechanism of RING and HECT ligases to induce ubiquitination (13–15). The removal of ubiquitin by deubiquitinating enzymes makes ubiquitination a highly dynamic process (16).

The E3 ubiquitin ligase family members have a complex and diverse role in the regulation of many inflammatory and metabolic processes that drive atherosclerosis. For example, the RING E3 ligase membrane-associated RING-CH-type finger 1 (MARCH1) limits the number of circulating inflammatory monocytes and hampers atherogenesis, whereas the HECT E3 ligase neural precursor cell-expressed developmentally down-regulated gene 4 (NEDD4-1) limits cholesterol efflux from macrophages, which promotes foam cell formation and atherosclerosis (17, 18). E3 ligases also have an important role in the regulation of the balance between T-cell activation and T-cell tolerance (13). By regulating the expression of the TCR, immune checkpoint proteins, cytokine receptors, and/or their downstream signaling pathways, E3 ligases determine the threshold for T-cell activation (Figure 1B) (10, 13–15). Interestingly, the E3 ligases Cbl-B, Itch, and GRAIL are upregulated during T-cell anergy, a hypo-responsive state in which immune cells do not acquire full effector functions, indicating that these enzymes act as natural inhibitors of T-cell activation (12, 13).

## Cbl-B as a Regulator of Immune Cell Activation

The RING-type E3 ubiquitin ligase Cbl-B is highly expressed in CD4^+^ and CD8^+^ T-cells in peripheral lymphoid tissues (15). Immune checkpoint proteins have a key role in the regulation of Cbl-B expression in these cells. Whereas, co-stimulatory molecules promote the degradation of Cbl-B, co-inhibitory molecules induce Cbl-B expression, thereby regulating various pathways that amplify T-cell activation, such as TCR- and cytokine-induced signaling (Figure 1B) (15). Cbl-B not only limits the steady-state expression of the TCR, but also promotes its downregulation upon antigen-mediated activation (19). Additionally, Cbl-B promotes ubiquitination-mediated degradation of early downstream signaling proteins of the TCR, such as protein kinase C (PKC) θ and phospholipase C (PLC) γ (15, 20). Loss of Cbl-B also uncouples the requirement for CD28-mediated co-stimulation for T-cell activation, which lowers the activation threshold of these cells (21–23). In addition to this, Cbl-B limits the production of IL2 and inhibits MAPK/ERK-mediated signaling pathways downstream of the IL2 receptor, thereby reducing the proliferation of these cells (23).

The functional relevance of Cbl-B in the regulation of T-cell activation is supported by observations in *Cbl-B*^−/−^ mice. Genetic deficiency of Cbl-B results in a hyper-responsive T-cell phenotype, characterized by increased proliferation and production of cytokines and granzymes, which triggers spontaneous autoimmune phenomena, e.g., T-cell dependent auto-antibody production and lymphocyte infiltration in various organs, and increases the susceptibility to experimental auto-immune diseases, such as experimental autoimmune encephalomyelitis (24, 25). Decreased expression of Cbl-B also enhances the effector functions of CD4^+^ T-cells and reduces the suppressive capacity of regulatory T-cells in patients with systemic lupus erythematosus and multiple sclerosis, indicating that Cbl-B is a clinically relevant negative regulator of T-cell driven inflammation (26, 27).

In addition to its role in the regulation of T-cells activity, Cbl-B also affects other immune cells. For example, genetic deficiency of Cbl-B increased B-cell proliferation and elicited T-cell-independent (auto)antibody production, possibly due to increased susceptibility to CD40-induced co-stimulation and enhanced Irf4-, NFκB-, and JNK-mediated signaling pathways (28, 29). In monocytes and macrophages, Cbl-B limits LFA1-ICAM1-mediated recruitment of monocytes toward inflamed tissues by inhibiting phosphorylation of the β2-chain of LFA1 (30). Cbl-B also promotes ubiquitination of TLR4, which hampers LPS-induced activation of MyD88 and degradation of IκBα, which enhances NFκB dependent inflammatory pathways in myeloid cells and ameliorates sepsis-induced macrophage-mediated lung inflammation (30–32). Together, these studies indicate that Cbl-B is a critical regulator of both adaptive and innate immune responses.

## The Protective Role of Cbl-B in Atherosclerosis

Cbl-B is predominantly expressed in T-cells and macrophages within human and murine atherosclerotic plaques (33). In human atherosclerotic plaques, Cbl-B expression negatively correlates with necrotic core size, which indicates a possible role for Cbl-B in hampering the progression of atherosclerosis. This role was confirmed in murine studies where genetic deficiency of Cbl-B aggravated atherosclerosis in *Apoe*^−/−^ mice (33). During the initial stages of atherogenesis, absence of Cbl-B increased the expression of chemokine receptors on monocytes, including CCR1, CCR2, and CCR7, which enhanced the recruitment of these cells to sites of vascular inflammation and increased macrophage abundance in the plaque (33). Deficiency of Cbl-B also increased CD36-mediated lipid uptake in bone marrow-derived macrophages, which promoted foam cell formation, and enhanced LPS-induced production of inflammatory mediators, such as TNF and IL6, and reactive oxygen species, indicating that Cbl-B deficiency induced an atherogenic monocyte and macrophage phenotype (33).

**Figure 2:**
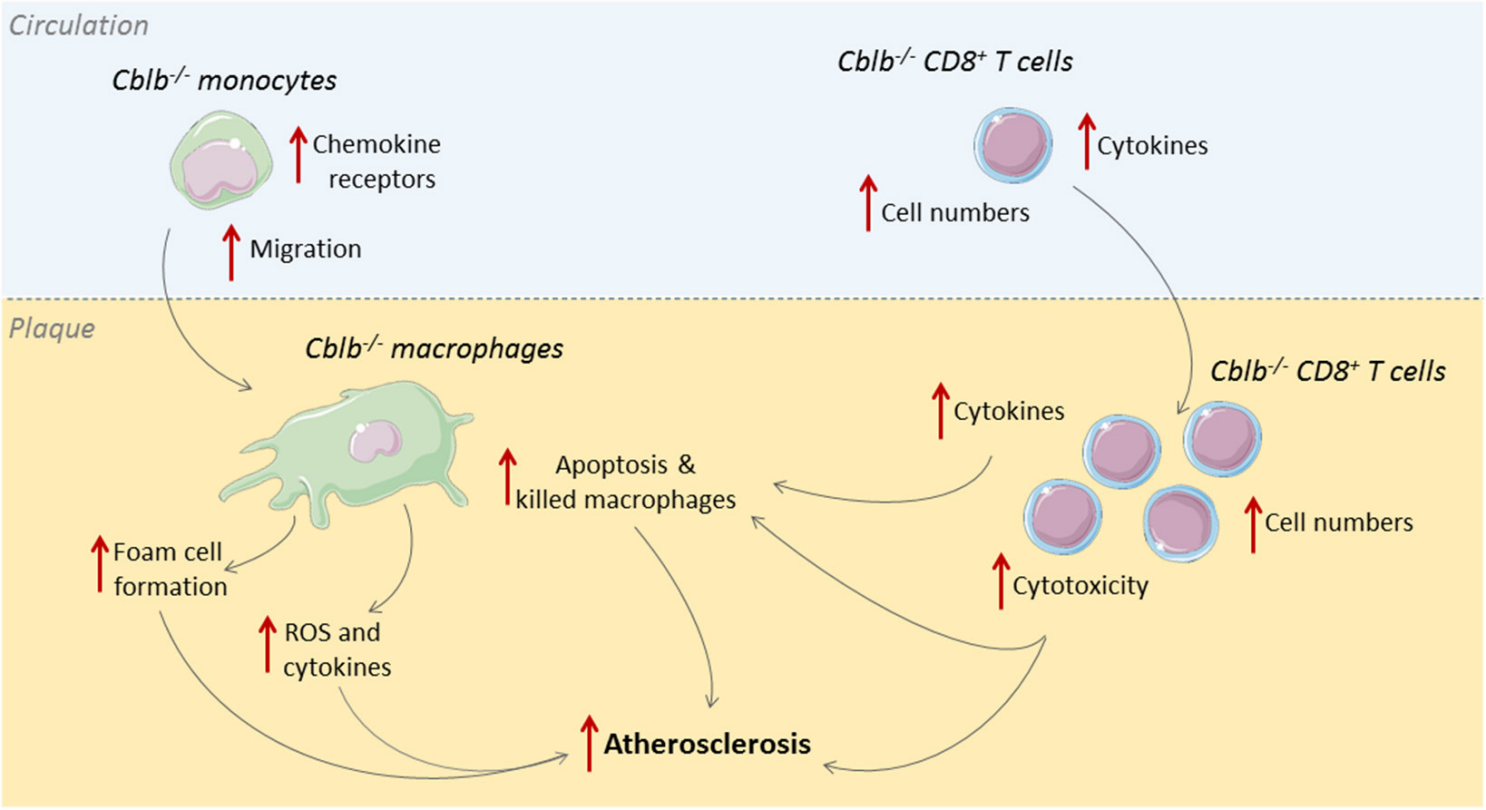
Cbl-B limits CD8^+^ T-cell and macrophage driven inflammation in atherosclerosis. During the initial stages of atherosclerosis, Cbl-B deficiency increases lesion formation by enhancing monocyte influx into the arterial wall; these monocytes subsequently develop into macrophages with an atherogenic phenotype (33). During the advanced stages of atherosclerosis, CBL-B deficiency increases plaque CD8^+^ T cell abundance and increases the cytotoxic phenotype of these cells, which aggravates plaque inflammation and provokes macrophage death, thereby enhancing the progression of plaques toward clinically unfavorable high-risk plaques with large necrotic cores (33).

When atherosclerosis progresses, adaptive immune cells, especially T-cells, are recruited to the plaque (34). Deficiency of Cbl-B increased the number of CD8^+^ T-cells in the circulation, spleen, and atherosclerotic plaques of *Apoe*^−/−^ mice by increasing the production of IL2 by these cells (33). Additionally, *Cblb*^−/−^ CD8^+^ T-cells were more resistant to apoptosis and less susceptible to suppression by regulatory T-cells (33). Cbl-B deficiency reduced CD44^−^CD62L^+^ naïve CD8^+^ T-cells and increased CD44^+^CD62L^+^ central memory CD8^+^ T-cells, and these cells produced more effector molecules, reflecting an activated cytotoxic T-cell phenotype (33). During the later stages of atherosclerosis, *Cblb*^−/−^ CD8^+^ T-cells provoked macrophage death in the plaque, resulting in increased necrotic core formation, which promoted plaque progression toward clinically unfavorable lesions (33). Although Cbl-B is also expressed in non-hematopoietic cells, such as smooth muscle cells, CD8^+^ T cell depletion studies in hematopoietic *Cblb*^−/−^*Apoe*^−/−^ mice confirmed that the progression of atherosclerosis in *Cblb*^−/−^ mice was predominantly driven by CD8^+^ T-cells (33). Together these data show that Cbl-B puts a brake on cytotoxic T-cell responses during atherogenesis, thereby limiting plaque inflammation and progression (Figure 2). Enhancing the activity Cbl-B may therefore be a therapeutic strategy for atherosclerosis.

## Itch: A Metabolic Regulator of Atherosclerosis

Itch, a HECT E3 ubiquitin ligase, has an important role in the regulation of T-cell activation and loss-of-function mutations in its ubiquitin ligase domain trigger a multisystem autoimmune disease in mice and men (35–39). Various studies in *Itch*^−/−^ mice identified a regulatory role in T-cell activation (40). For example, upon TCR-mediated activation, Itch induces the ubiquitination-mediated degradation of TCR signal transduction molecules, including PLCγ, PKCθ, and zeta-chain-associated protein kinase (ZAP) 70, thereby increasing the threshold for T-cell activation (36, 41). Itch also promotes the ubiquitin-mediated degradation of the IL2 receptor signaling intermediate Janus kinase 1, which limits T-cell proliferation. Effector functions of CD4^+^ T-cells are further hampered by the Itch-mediated degradation of the transcription factor JunB, which halts Th2 polarization (42). Additionally, Itch promotes FoxP3 expression in CD4^+^ T-cells by facilitating the ubiquitination-mediated activation of TGFβ inducible early gene-1 (TIEG1), resulting in increased development of regulatory T-cells (43).

Despite its important role in the regulation of T-cell activation and regulatory T-cell development, genetic deficiency of Itch reduced atherosclerotic lesion size in *Apoe*^−/−^ mice by ~75% (44). Although this study did not include a detailed analysis of T-cell populations in the plaque, circulation or lymphoid tissues, a striking metabolic phenotype, characterized by a ~50% decrease in plasma cholesterol levels, was observed in *Itch*^−/−^*Apoe*^−/−^ mice (44). The lower cholesterol levels resulted from an increased expression of key lipid metabolism regulators, including SIRT6 and SREBP2, which enhanced low density lipoprotein receptor-mediated cholesterol uptake in the liver (44). In contrast to full body knock-out mice, hematopoietic deficiency of Itch did not affect cholesterol levels or atherosclerosis in *Apoe*^−/−^ mice, indicating that the atheroprotective effect of Itch depended on the atheroprotective lipid profile (44). Although it would be interesting to investigate T-cell populations and activity in atherosclerotic *Itch*^−/−^ mice, the current data suggest that therapeutic modulation of Itch will not be a suitable strategy to target the residual inflammatory risk in patients with CVD.

## Grail: A Regulator of Immune Cell Activation in Atherosclerosis?

The RING-type E3 ubiquitin ligase GRAIL is well-known for its role in the regulation of T-cell activity (12, 13, 45). Genetic deficiency of GRAIL increases the expression of the TCR and CD3 on T-cells and *Grail*^−/−^ T-cells do not depend on co-stimulatory signals for their activation (45). Following TCR-mediated activation, GRAIL promotes the ubiquitination-mediated degradation of the endocytosolic TCR-CD3 complex and restricts the expression of the transcription factor nuclear factor of activated T-cells (NFATc1), thereby limiting T-cell activation, IL2-induced proliferation, and the expression of Th1 (IFNγ) and Th17 (IL17, IL21, IL22) cytokines (45). In accordance with the negative regulatory role of GRAIL in T-cell activation, aged *Grail*^−/−^ mice are more susceptible to experimental auto-immune diseases and spontaneously develop autoimmune phenomena, e.g., immune cell infiltration in organs and auto-antibody formation (45). Adoptive transfer of *Grail*^−/−^ CD4^+^ T-cells into lymphocyte deficient *Rag1*^−/−^ mice aggravated experimental autoimmunity, indicating that the autoimmune phenotype of *Grail*^−/−^ mice was predominantly driven by aberrant CD4^+^ T-cell responses (45). In accordance, polymicrobial sepsis in mice induces the upregulation of GRAIL in CD4^+^ T-cells, which limits their proliferation and effector functions and contributes to the T-cell immunoparalysis that is common in sepsis (46, 47).

Whether GRAIL has a similar immunomodulatory role in atherosclerosis is currently unknown, but several lines of evidence implicate a role for this E3 ligase in atherogenesis. First, GRAIL regulates the expression of co-stimulatory and co-inhibitory molecules on T-cells, which determines the threshold for T-cell activation and steers T-cell effector functions (10, 48). For example, GRAIL promotes ubiquitination-mediated degradation of the co-stimulatory molecules CD40L, OX-40, and CD137 in CD4^+^ and CD8^+^ T-cells and enhances the expression of the co-inhibitory molecules cytotoxic T-lymphocyte–associated antigen 4 (CTLA4) and glucocorticoid-induced TNFR-related protein (GITR) on T-cells, which resulted in an anti-inflammatory immune checkpoint protein landscape that limits the activation of these cells (48–51). Additionally, *Grail*^−/−^ CD8^+^ cytotoxic T-cell exhibit an increased migratory potential and enhanced production of IFNγ and granzyme B, which promote the progression of atherosclerosis (33, 51). Finally, GRAIL enhances regulatory T-cell responses by increasing FoxP3 and TGFβ expression in CD4^+^ T-cells, which may boost the protective role of regulatory T-cells in atherogenesis (45, 49). Together, these data implicate a protective role for GRAIL in atherosclerosis by limiting effector T-cell responses and enhancing the anti-inflammatory effects of regulatory T-cells, a concept that should be addressed in future studies.

## The Therapeutic Potential of E3 Ubiquitin Ligases

Insights in the anti-inflammatory potential of E3 ligases in humans come from observations in patients with leprosy, an infectious disease caused by *Mycobacterium leprae*, which is associated with a hyporesponsive T-cell phenotype (52). An 80% increase in Cbl-B expression is observed in circulating CD4^+^ and CD8^+^ T-cells of leprosy patients, which enhances the expression of the co-inhibitory molecule CTLA4 and downregulates CD28, thereby increasing the threshold for T-cell activation (52, 53). Moreover, these cells produce less IL2, which further limits T-cell-mediated immunity against *M. leprae* (53, 54). Similarly, *Trypanosoma cruzi, Mycobacterium tuberculosis*, and Helminth infections are associated with severe CD4^+^ T-cell hyporesponsiveness due to increased expression of GRAIL (55–57). Phenotypically, T-cells that upregulate GRAIL during an infection have an exhausted, anti-inflammatory phenotype, characterized by high expression of CTLA4 and programmed cell death protein 1 (PD1), and low IFNγ and IL2 production (55). Although immunological responses in patients with infectious diseases undoubtedly differ from the inflammatory response in patients with atherosclerotic CVD, these data at least indicate that increased expression of Cbl-B and GRAIL limits the inflammatory propensity of T-cells in humans.

Enhancing T-cell effector functions is a potent anti-cancer therapy and CRISPR screens of human T-cells identified Cbl-B as a primary candidate to boost their activity (58–60). Accordingly, adoptive transfer of *Cbl-B*^−/−^ CD8^+^ T-cells improved anti-tumor immunity in various models (61–63). Interestingly, siRNA-mediated silencing of Cbl-B in CD8^+^ T-cells also improved the efficacy of dendritic cell-based tumor vaccines, indicating that inhibition of Cbl-B may become an adjuvant strategy to improve the efficacy of cancer immunotherapy (64–66). Whether enhanced activity of Cbl-B is associated with increased tumorigenesis is currently unknown and should be investigated in future studies.

As with any anti-inflammatory intervention, immune suppression is a potential concern. A specific concern of strategies that enhance Cbl-B activity is candidiasis, as experimental studies demonstrated that a deficiency of Cbl-B improved survival rates in mice subjected to *Candida albicans* infection (67–69). As the beneficial effects of Cbl-B predominantly depended on improved myeloid-driven antifungal immune responses, cell type-specific modulation of E3 ligases, for example by antibody-drug conjugates that target T-cells, may circumvent these potential immunosuppressive side effects (67–69). Another potential concern is the complex role of Cbl-B in anti-viral immunity. For example, Cbl-B limits excessive immune activation in mice that are infected with an intermediate dose of lymphocytic choriomeningitis virus (LCMV), which improves their survival (15, 70, 71). On the other hand, increased Cbl-B activity promotes the entry of the hepatitis C virus (HCV) into hepatocytes and contributes to the development of HCV-related autoimmunity by enhancing the formation of anergic B cells (72, 73). Future studies should therefore scrutinize the role of E3 ligases in antimicrobial immunity before strategies that enhance the function of these enzymes are developed toward the clinic.

## Future Directions

Pharmacological strategies to enhance the activity of Cbl-B or GRAIL do currently not exist, but several strategies can be applied to boost the activity of these E3 ligases. For example, small molecule-mediated blockage of intramolecular inhibitory regions, such as the unphosphorylated N-terminal region of Cbl-B that covers the E2 ligase binding site of the RING domain, can be used to enhance the activity of Cbl-B (74). Additionally, small molecules or peptides that support the open and active conformation of Cbl-B may increase its activity. Alternatively, natural inhibitors of E3 ligases can be targeted to enhance their function. For example, upon TCR-mediated activation, Src homology region 2 domain-containing phosphatase-1 (SHP-1) binds to Cbl-B, which abolishes its ubiquitin ligase activity, suggesting that inhibition of this interaction may enhance Cbl-B activity, a concept that should be investigated in future studies (75).

## Conclusions

Given the abundant presence of activated T-cells in human atherosclerotic lesions, modulation of T-cell activity is a promising strategy to target the residual inflammatory risk in patients with atherosclerotic CVD. Although the number of studies on the role of Cbl-B and GRAIL in experimental atherosclerosis and human atherosclerotic CVD is limited, these findings, in conjunction with studies from the immunological field, identify Cbl-B and GRAIL as natural brakes on T-cell activation, increasing the expression and/or activity of these E3 ubiquitin ligases may therefore be an attractive strategy to temper T-cell driven inflammation in atherosclerotic CVD.

## Author Contributions

TS wrote the first draft of the manuscript. TS, KP, and WV wrote the sections of the manuscript. All authors contributed to manuscript revision, read, and approved the submitted version.

## Conflict of Interest

The authors declare that the research was conducted in the absence of any commercial or financial relationships that could be construed as a potential conflict of interest.
